# Effect of Platelet-activating Factor on *in vitro* and *in vivo* Interleukin-6 Production

**DOI:** 10.1155/S0962935194000384

**Published:** 1994

**Authors:** B. Pignol, T. Maisonnet, P. Guinot, J. M. Mencia-Huertac, P. Braquet

**Affiliations:** Institut Henri Beaufour Department of Immunology 1 avenue des Tropiques Les ULIS 91952 France

## Abstract

The aim of the present study was to investigate the possible effect of platelet-activating factor (PAF), by comparison with interleukin-1β and polyriboinositic/polyribocytidylic (poly I–C) acid, on IL-6 production by L 929 mouse fibroblasts. At concentrations above 1 μM PAF, the production of IL-6 by mouse fibroblasts was enhanced in a dose dependent fashion. At 5 μM PAF, the peak increase (60.1 ± 19.4 U/ml) was similar to that induced by 50 μg/ml poly I–C (60.0 ± 35.0 U/ml) and higher than the one evoked by 100 U/ml IL-1β (3.8 ± 1.8 U/ml). The increase of 11-6 activity induced by 5 μM PAF was maximal after a 22 h incubation period with L 929 cells. Lyso-PAF (5 μM) also increased IL-6 activity from fibroblasts to a similar extent compared with 5 μM PAF. In addition, the IL-6 activity induced by 5 μM PAF was still observed when the specific PAF antagonist, BN 52021 (10 μM), was added to the incubation medium of L 929 cells. The result suggests that the production of IL-6 by L 929 cells evoked by PAF *in vitro* is not receptor mediated. The *in vivo* effect of PAF on IL-6 production was also investigated in the rat. Two hours after intravenous injection of PAF (2 to 4 μg/kg), a dramatic increase of IL-6 activity in rat serum was observed, this effect being dose dependent. The increase of IL-6 induced by 3 μg/kg PAF was not observed when the animals were treated with the PAF antagonist, BN 52021 (1 to 60 mg/kg0. These results demonstrate that PAF modulates IL-6 production and that the *in vivo* effect is receptor mediated.


Mediators of Inflammation 3, 281–285 (1994)

### Effect of platelet-activating factor on *in vitro* and *in vivo* interleukin-6 production

B. Pignol, T. Maisonnet, P. Guinot, J. M. Mencia-Huerta[CA] and P. Braquet

Institut Henri Beaufour, Department of Immunology, 1 avenue des Tropiques, 91952 Les ULIS, France

[CA]Corresponding Author

**The aim of the present study was to investigate the possible effect of platelet-activating factor (PAF), by comparison with interleukin-1β and polyriboinositic/polyribocytidylic (poly I–C) acid, on IL-6 production by L 929 mouse fibroblasts. At concentrations above 1 μM PAF, the production of IL-6 by mouse fibroblasts was enhanced in a dose dependent fashion. At 5 μM PAF, the peak increase (60.1 ± 19.4 U/ml) was similar to that induced by 50 μg/ml poly I–C (60.0 ± 35.0 U/ml) and higher than the one evoked by 100 U/ml IL-1β (3.8 ± 1.8 U/ml). The increase of IL-6 activity induced by 5 μM PAF was maximal after a 22 h incubation period with L 929 cells. Lyso-PAF (5 μM) also increased IL-6 activity from fibroblasts to a similar extent compared with 5 μM PAF. In addition, the IL-6 activity induced by 5 μM PAF was still observed when the specific PAF antagonist, BN 52021 (10 μM), was added to the incubation medium of L 929 cells. The result suggests that the production of IL-6 by L 929 cells evoked by PAF *in vitro* is not receptor mediated. The *in vivo* effect of PAF on IL-6 production was also investigated in the rat. Two hours after intravenous injection of PAF (2 to 4 μg/kg), a dramatic increase of IL-6 activity in rat serum was observed, this effect being dose dependent. The increase of IL-6 induced by 3 μg/kg PAF was not observed when the animals were treated with the PAF antagonist, BN 52021 (1 to 60 mg/kg). These results demonstrate that PAF modulates IL-6 production and that the *in vivo* effect is receptor mediated.**

**Key words:** Interleukin-1β, Interleukin-6, Mouse fibroblasts, Platelet-activating factor

### Introduction

A T cell line derived, B cell differentiation factor (B cell stimulatory factor-2)[1] has been identified in both human and mouse.[2] This protein has been shown to be identical to fibroblast derived interferon-$\beta_2$ (IFN-$\beta_2$)[3,4] and has been referred to as interleukin-6 (IL-6).[5] This cytokine is produced by a wide variety of cell types and demonstrates many biological effects, both *in vitro* and *in vivo*.[6] Although its primary source appears to be the monocyte/macrophage,[7,8] IL-6 is also induced in human and murine fibroblasts on stimulation with IL-1 and tumour necrosis factor (TNF).[4,9] Biological activities of IL-6 include induction of acute phase protein synthesis by hepatocytes,[10] stimulation of immunoglobulin synthesis by B cells,[6] increase of T lymphocyte responses,[11,12] and proliferation of multi-potential haematopoietic progenitors in synergy with IL-3.[13]

It has been demonstrated previously that the phospholipid mediator, PAF modulates the production of the monocyte/macrophage derived cytokine, IL-1, both *in vitro*[14] and *in vivo*.[15] Indeed, this lipid mediator either increases or decreases IL-1 activity in cell supernatants from lipopolysaccharide (LPS) stimulated rat macrophages according to the dose and the time period of addition. As accessory cells produce both IL-1[16] and IL-6,[7] the question arose whether PAF could also modulate the *in vitro* and *in vivo* production of IL-6.

### Materials and Methods

*Reagents:* The double-stranded RNA, polyriboinosinic/polyribocytidylic (poly I–C) and bovine serum albumin (BSA) were purchased from Sigma (St Louis, MO, USA). IL-1β was obtained from TEBU (Les Ulis, France). IL-6 was obtained from Immugenex (Los Angeles, CA, USA). Fibroblasts were cultured in DMEM (Flow Laboratories, Virginia, USA) containing 10% foetal bovine serum (FBS, Gibco, Paisley, UK). In the *in vitro* experiments, BN 52021[17] (IHB Research Laboratories, Le Plessis Robinson, France) was dissolved in dimethylsulfoxide (DMSO) at the 10 mM concentration and further diluted in a 10mM HEPES solution. For the *in vivo* experiments the drugs were dissolved in physiological serum containing 0.25% BSA. PAF (C16-PAF) and lyso-PAF were obtained from Novabiochem (Clery en vexin, France).

© 1994 Rapid Communications of Oxford Ltd

*Cell cultures:* The mouse embryonic fibroblast cell line, L 929, was cultured in DMEM containing 10% FBS. The cells ($2.5 \times 10^5$/10 ml) were incubated at 37°C in multi-well dishes for about 1 week, i.e. a time period at which the cells reach confluence. At the end of this incubation period, the medium was removed and 5 ml of fresh medium was added. Various substances, namely IL-1β, poly I–C, lyso-PAF, PAF, BN 52021 or the vehicle of PAF, i.e. DMEM containing 0.25% BSA, or DMSO, were added at defined concentrations or dilutions. In some experiments, after incubation at 37°C for 4 h to 48 h as specified, the cell suspensions were centrifuged at 400 × ***g*** for 10 min and the supernatants were collected and stored at –20°C prior to assay for IL-6 activity. No effect of the solvent of PAF or DMSO on the IL-6 release was noted (data not shown). In some experiments, the release of lactate dehydrogenase (LDH) was assessed using commercial kits from Boehringer (Mannheim, Germany).

In vivo *experiments:* Sprague–Dawley rats (250 g) were injected intravenously with either PAF or lyso-PAF and blood samples were obtained after 1, 2, 3, 4, 6 and 24 h. In some experiments, BN 52021 or solvent alone was injected intraperitoneally 30 min prior to the injection of PAF or vehicle.

*Bioassay of IL-6:* IL-6 activity in cell-free supernatants from mouse fibroblasts or rat serum was measured by the proliferation of the 7DT1 cell line (obtained through the courtesy of Dr J. Van Snick, Brussels, Belgium). Briefly, triplicates of $2 \times 10^3$ cells in 0.2 ml were added with defined dilutions of the supernatants or sera to be tested. Appropriate control media or known amounts of rIL-6 were also included. In some experiments, the anti-IL-6 antibody, 6B4 (obtained through the courtesy of Dr J. Van Snick, Brussels, Belgium), was added to supernatants from L 929 for 30 min prior to the assessment of IL-6 activity. After defined incubation periods, the number of viable cells was evaluated by the colorimetric determination of the levels of β-hexosaminidase. The data are expressed as means ± S.E.M. of the indicated number of experiments and the results were assessed for statistical significance using the analysis of variance. Results are either expressed in optical density (OD) or in equivalent U/ml of recombinant IL-6 (rIL-6), with respect to the linear portion of calibration curves performed with known amounts of the recombinant cytokine.

In a preliminary experiment, as measured by the colorimetric determination of β-hexosaminidase, the proliferation of 7TD1 was shown to be dependent on both the dose of rIL-6 and the incubation period. A minimal cell proliferation, although not dose related, was observed after 48 h. After 72 h, the increase in the β-hexosaminidase content of the cells was related to IL-6 concentration and reached plateau values after 96 h. At this later time period, a dose related effect was noted in the range of concentrations from 0.01 U/ml to 5 U/ml rIL-6 (data not shown), a plateau being reached for concentrations above 5 U/ml. Since a maximal increase in the β-hexosaminidase content was noted after 96 h, this time period was used to assess the IL-6 activity in the various supernatants.

### Results

*Effect of PAF on IL-6 production by L 929 cells:* When L 929 cells were incubated for 48 h at 37°C in medium alone, low IL-6 activity was measured in the supernatants. A 96 h incubation period was demonstrated previously to be optimal for the production of IL-6 by this cell type and was thus used.[8] The activity in the supernatants of unstimulated L 929 cells decreased with the dilution from 1/2 to 1/256 (data not shown). When the results are expressed in U/ml, L 929 cells produced 1.3 ± 0.7 U/ml. Addition of IL-1β (100 U/ml) to L 929 cultures increased the IL-6 section, although not to a significant level at the 1/2 dilution or below the 1/8 one. In contrast, when the supernatants were measured at 1/4 and 1/8 dilutions, IL-1β significantly enhanced the IL-6 production by 102% and 96%, respectively (data not shown). Poly I–C (50 μg/ml) markedly increased IL-6 production with a higher efficiency compared with IL-1β (Fig. 1). In a preliminary set of experiments, the effect of the phospholipid mediator, PAF, was compared with one of the two agonists and shown to enhance markedly the IL-6 production from L 929 cells at the 5 μM concentration (Fig. 1). Lower concentrations of PAF were also used (1 μM to 10 fM), but no effect on IL-6 production was noted.

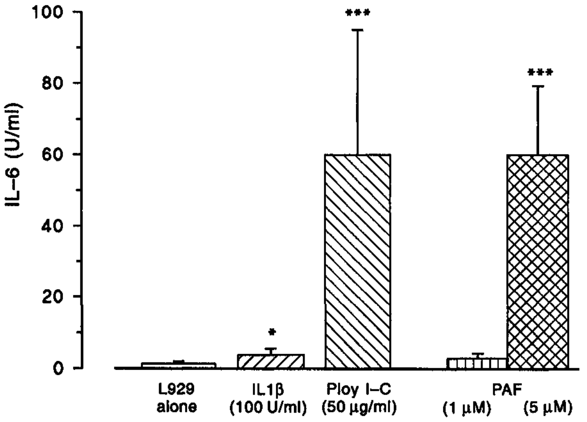

FIG. 1. IL-6 activity in supernatants from cultured L 929 cells incubated with various agonists. Fibroblasts (1 week old) were cultured in the presence or absence of IL-1β (100 U/ml), poly I–C (50 μg/ml) or PAF (1 μM and 5 μM) for 48 at 37°C. At the end of the culture period, the supernatants were collected and assayed for IL-6 activity, as measured by the colorimetric determination of β-hexosaminidase which reflects the proliferation of 7TD1 cells. Results are expressed in equivalent U/ml of recombinant IL-6 calculated from a standard curve established with known amounts of this cytokine and as means ± S.E.M. of four experiments. $^*p < 0.05$; $^{**}p < 0.01$.

The IL-6 activity in the various supernatants was calculated as equivalent U/ml with respect to calibration curves performed with known amounts of rIL-6. When the results are expressed in U/ml, PAF at the 5 μM concentration and poly I–C at the 50 μg/ml, a marked release of IL-6 from L 929 fibroblasts (60.1 ± 19.4 U/ml) and (60.0 ± 35 U/ml), respectively, was induced compared with 100 U/ml IL-1β (3.8 ± 1.8 U/ml). In contrast, 1 μM PAF had no significant effect (2.9 ± 1.4 U/ml) on IL-6 production compared with unstimulated L 929 cells (1.3 ± 0.7 U/ml) (Fig. 1).

The kinetics of released and cell associated IL-6 activity upon stimulation of L 929 with 5 μM PAF and poly I–C (100 μg/ml) were then investigated. The spontaneous IL-6 activity was shown to be significantly increased after 22 h, 28 h and 48 h, compared with the value measured after 4 h. A significant enhancement of the released IL-6 activity by PAF was observed after 22 h and up to 48 h following stimulation of L 929 cells, compared with the spontaneous production of this cytokine (Fig. 2). By comparison, poly I–C increased the released IL-6 activity as early as 4 h after addition of this agonist and up to 48 h. Neither, PAF nor poly I–C significantly modified cell associated IL-6 activity above basal level (0.099 ± 0.010 OD unit). To determine whether the activity inducing the proliferation of 7TD1 was indeed due to IL-6, supernatants and cell associated activity from L 929 cells stimulated or not with PAF or poly I–C were collected at defined time intervals and incubated with the anti-IL-6 antibody (1/160 dilution) for 30 min prior to adding to 7TD1 cells for 96 h. The optical density measured at the end of the 96 h incubation period in supernatants when the anti-IL-6 antibody was added, was identical to the one observed upon incubation of 7TD1 in the absence of IL-6 (0.063 ± 0.012, OD unit, i.e. < 0.4 U/ml, $n = 7$).

The number of cells alive counted with trypan blue was identical after treatment with PAF and poly I–C compared with unstimulated L 929 cells (Table 1), indicating that the increase of IL-6 activity in supernatants of PAF or poly I–C stimulated cells was not due to a cytotoxic effect of these compounds. In addition, the lack of cell cytoxicity of 5 μM PAF was confirmed by the measurement of lactate dehydrogenase (LDH) in supernatants. In contrast, 100 μg/ml poly I–C was toxic for L 929 cells after 48 h in culture (115.2 ± 28.8 U/ml LDH compared with 54.5 ± 15.1 U/ml in controls).

In the next series of experiments, the dose dependent effect of PAF (1 μM to 5 μM) on the release of IL-6 activity from L 929 cells was investigated after 48 h. A dose related increase in IL-6 activity in the supernatants of cells stimulated with concentrations of PAF above 1 μM was indeed observed (Fig. 3). Addition of the anti-IL-6 antibody to supernatants from unstimulated and PAF stimulated L 929 cells inhibited by 84 to 90% the proliferation of 7TD1 cells (Fig. 3).

The specificity of the effect of PAF on the release of IL-6 activity from L 929 was investigated next. No specificity was observed in this *in vitro* model using mouse fibroblasts. Indeed, a similar IL-6 activity after 48 h incubation of L 929 cells with 5 μM lyso-PAF or 5 μM PAF was noted. When the results were calculated as equivalent units, lyso-PAF and PAF produced after 48 h 77.1 ± 36.5 U/ml and 66.1 ± 17.0 U/ml IL-6, respectively, compared with unstimulated L 929 cells (2.0 ± 0.7 U/ml). Supernatants from L 929 cells treated with lyso-PAF and PAF incubated with anti-IL-6 antibody did not exhibit significant IL-6 activity (97% inhibition). In addition, BN 52021 (10 μM) did not interfere with the enhancing effect of PAF on the IL-6 activity produced by L 929 cells after 48 h (data not shown).

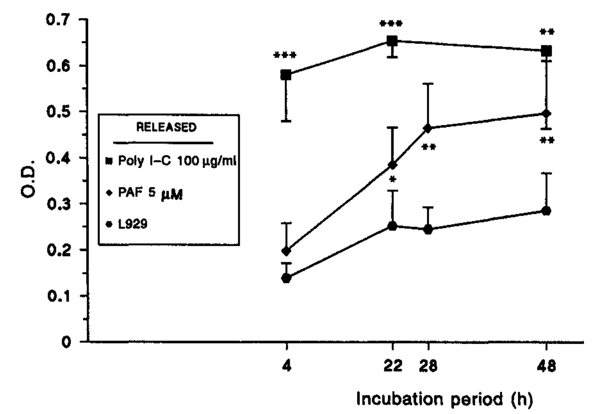

FIG. 2. Kinetics of the released IL-6 activity after incubation of L 929 cells with various agonists. Fibroblasts (1 week old) were cultured in the presence or absence of poly I–C (100 μg/ml) or PAF (5 μM) for 4, 22, 28 and 48 h at 37°C. At each indicated time point, the supernatants were collected and assayed for IL-6 activity. In these experiments the supernatants were analysed at the 1/4 dilution. Results are expressed as O.D. units and as means ± S.E.M. of four experiments. *$p < 0.05$; **$p < 0.01$; ***$p < 0.001$.

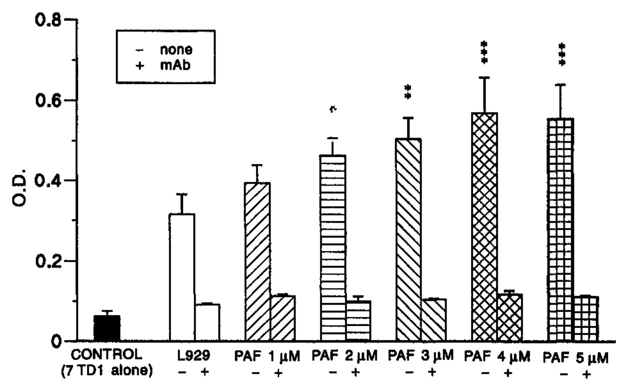

FIG. 3. Dose dependent effect of PAF on IL-6 production by L 929 cells. IL-6 activity in supernatants from L 929 cells incubated with defined concentrations of PAF. Fibroblasts (1 week old) were cultured in the presence or in the absence of PAF (1 μM to 5 μM) for 48 h at 37°C. At the end of the culture period, the supernatants were collected, the monoclonal antibody (mAb) directed against IL-6 added or not as appropriate, and assayed for IL-6 activity. In these experiments the supernatants were analysed at the 1/4 dilution. Results are expressed as O.D. and as means ± S.E.M. of five experiments. *$p < 0.05$; **$p < 0.01$; ***$p < 0.001$.

**Table 1.** Time dependent effect of incubation of L 929 cells in the presence of poly I–C and PAF on cell viability

| | Cell number ($10^6$/ml) | | | |
|---|---|---|---|---|
| | 4 h | 22 h | 28 h | 48 h |
| L 929 | 8.5 ± 0.9 | 7.6 ± 0.5 | 8.7 ± 1.5 | 10.7 ± 1.3 |
| Poly I–C (100 μg/ml) | 7.1 ± 0.5 | 8.1 ± 1.0 | 9.9 ± 1.1 | 10.9 ± 1.2 |
| PAF (5 μM) | 8.9 ± 1.0 | 7.8 ± 1.0 | 10.3 ± 1.4 | 12.4 ± 1.9 |

Fibroblasts (1 week old) were cultured in the presence or absence of poly I–C (100 μg/ml) or PAF (5 μM) for 4, 22, 28 and 48 h at 37°C. At each indicated time point, the number of cells alive was determined after addition of trypan blue. Results are expressed as means ± S.E.M. of four experiments.

*Effect of PAF on IL-6 levels in rat serum:* Intravenous injection of lyso-PAF at 2 and 3 μg/kg did not modify the level of IL-6 in rat serum after 1 h to 24 h (Fig. 4). In contrast, the IL-6 level was markedly increased in a dose dependent fashion when rats were treated with PAF at doses ranging from 2 to 4 μg/kg and with a peak effect observed 2 h after injection (Fig. 4). Indeed, treatment with 2, 3 and 4 μg/kg PAF increased by 44-, 63- and 73-fold the level of IL-6 in rat serum, respectively. However, intravenous injection of PAF at 4 μg/kg was toxic, since half of the rats died during the first hour of experimentation. Although the maximum increase obtained after 2 h was of lower intensity with 3 μg/kg PAF compared with 4 μg/kg, this dose of PAF and this time period were chosen for further experiments investigating the effect of BN 52021 on the *in vivo* IL-6 production.

Intraperitoneal injection of BN 52021 at 0.1, 1, 5 and 15 mg/kg was performed 30 min before intravenous injection of 3 μg/kg PAF or vehicle alone. No effect of treatment of the animals with BN 52021 on the basal level of IL-6 measured at 2 h was noted. When the rats were treated with the dose of 1, 5 or 15 mg/kg BN 52021, the peak of IL-6 in serum observed 2 h following intravenous injection of 3 μg/kg PAF was not observed ($p$ < 0.001, Fig. 5). By contrast, the dose of 0.1 mg/kg was inefficient in preventing the PAF induced increase in IL-6 levels in rat serum.

In a second series of experiments, the possibility that treatment of the rat with BN 52021 delayed the appearance of IL-6 in serum was investigated. In these experiments, only the 15 mg/kg dose of BN 52021 was investigated. As presented in Fig. 6, no IL-6 activity in serum was detected at all time points of this kinetic study.

### Discussion

In the present study, it is confirmed that the fibroblast cell line, L 929, produces IL-6-like activity and that this production is enhanced by IL-1β and to a greater extent by poly I–C, PAF and lyso-PAF. IL-1β is already known as one of the physiologic inducers of IL-6 by fibroblasts. The double-stranded RNA, poly I–C, has also been shown to induce IL-6,

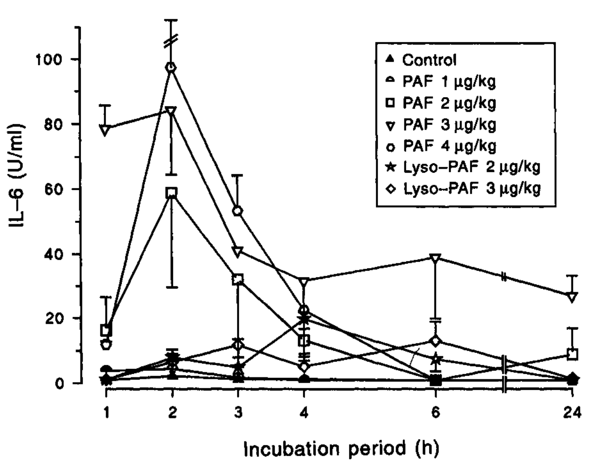

FIG. 4. Effect of intravenous injection of various compounds in rats. Sprague–Dawley rats (250 g) were injected intravenously with PAF or lyso-PAF and blood samples were obtained after defined time intervals. IL-6 in sera was determined as described in the Materials and Methods section. Results are expressed in equivalent U/ml Il-6 and as means ± S.E.M. of the three to five experiments.

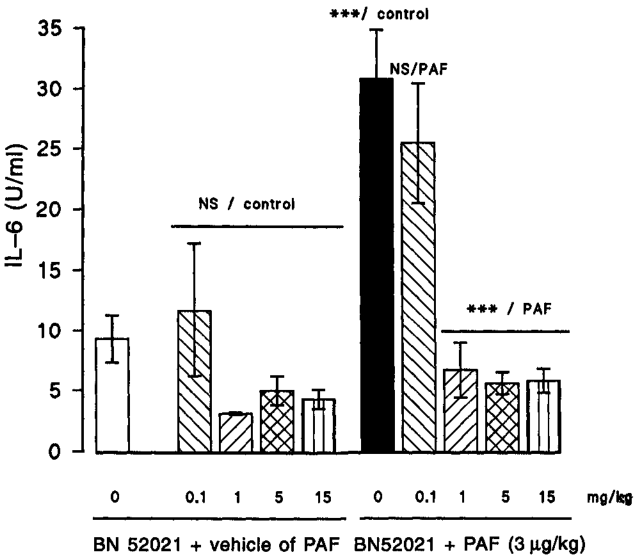

FIG. 5. Effect of BN 52021 on the *in vivo* production of IL-6 by PAF. Sprague–Dawley rats (250 g) were injected intravenously with PAF (3 μg/kg) or solvent of PAF. The animals were treated or not with BN 52021 (0.1 to 15 mg/kg) or vehicle 30 min prior to injection of PAF. Blood samples were obtained after 2 h and IL-6 in sera was determined as described in the Materials and Methods section. The results are expressed in equivalent U/ml IL-6 and as means ± S.E.M. of the three to six experiments. ***$p$ < 0.001.

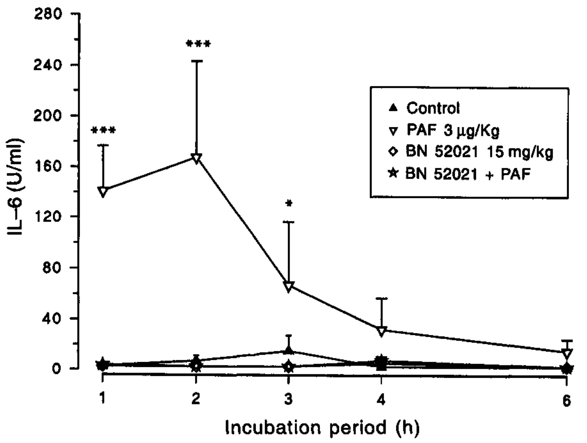

FIG. 6. Kinetic of the effect of BN 52021 on the *in vivo* production of IL-6 by PAF. Sprague–Dawley rats (250 g) were injected intravenously with PAF (3 µg/kg) or solvent of PAF. The animals were treated or not with BN 52021 (15 mg/kg) or vehicle 30 min prior to injection of PAF. Blood samples were obtained after a defined time interval and IL-6 in sera was determined as described in the Materials and Methods section. The results are expressed in equivalent U/ml IL-6 and as means ± S.E.M. of the three to five experiments. *$p < 0.05$; ***$p < 0.001$.

although more efficiently compared with IL-1β.[9] The results presented here demonstrate that L 929 cells stimulated with PAF or lyso-PAF produced high IL-6-like activity. The increase in IL-6 activity observed with lyso-PAF is puzzling and could be due to the presence of acetyl-CoA: 1-0-alkyl-*sn*-glyceryl-3-phosphorylcholine acetyltransferase in fibroblasts. However, this hypothesis, was not investigated in the present study. Alternatively, the possibility that lyso-PAF evokes IL-6 independently of the stimulation of a specific receptor is also open.

The fact that 10 µM BN 52021 did not inhibit the IL-6 production induced by 5 µM PAF was unexpected, although this lack of effect of this PAF antagonist might be due to an unfavourable antagonist/agonist ratio (2:1, on a molar basis). Higher doses of BN 52021 could not be investigated due to the toxic effect of the vehicle (DMSO) of this drug on L 929 cells. These results, as well as the induction of IL-6 release by lyso-PAF, indicate that the action of PAF could be unrelated to the stimulation of PAF specific receptors. The possibility that lyso-PAF could be the agonist implicated in the induction of IL-6 in L 929 cells is under investigation.

High levels of circulating IL-6 have been observed in various inflammatory states such as widespread burns[18] and septic shock.[19] Injection of PAF in rats evokes a dramatic increase in IL-6 activity in serum. The *in vivo* effect of PAF appears to be mediated via specific binding sites because BN 52021 prevents the PAF induced increase in IL-6 production. In addition, administration of lyso-PAF in rats is inefficient in inducing IL-6 production, further strengthening the specificity of the effect of PAF.

The present data demonstrate that PAF might play an *in vivo* immunoregulatory role via an increase of IL-6 level in serum. In fibroblasts, IL-6 appears to be an autocrine factor[20,21] and the release of this cytokine in response to mediators elaborated in areas of tissue damage may contribute to the local inflammatory response. These data support the concept that PAF is not only a mediator of acute allergic and inflammatory reactions but might also contribute to long-term processes such as chronic diseases.

### References

1. Hirano T, Yasukawa H, Harada T, *et al.* Complementary DNA for a novel interleukin that induces B lymphocytes to produce immunoglobin. *Nature* 1986; **324:** 73.7.
2. Van Snick J, Cayphas S, Vink A, Uyttenhove C, Coulie P, Simpson R. Purification and $NH_2$-terminal amino acid sequence of a T-cell derived lymphokine with growth activity for B-cell hybridomas. *Proc Natl Acad Sci* USA 1986; **83:** 9679.
3. Van Damme J, Cayphas S, Van Snick R, *et al.* Purification and characterization of human fibroblast derived hybridoma growth factor identical to T-cell derived B-cell stimulatory factor 22 (interleukin-6). *Eur J Biochem* 1987; **168:** 543.
4. Van Damme J, Opdenakker G, Simpson J, *et al.* Identification of the human 26 KD protein, interferon-beta-2 as a B-cell hybridoma/plastocytoma growth factor induced by interleukin-1 and tumor necrosis factor. *J Exp Med* 1987; **165:** 914.
5. Poupart P, Vandenabeele S, Cayphas J, *et al.* B-cell growth modulating and differentiating activity of recombinant human 26 KDa protein. *EMBO J* 1987; **6:** 1219.
6. Kishimoto T. B-cell stimulatory factors (BSFs): molecular structure, biological functions, and regulation of expression. *J Clin Immunol* 1987; **7:** 343.
7. Aarden L, De Groot E, Schaap O, Lansdorp P. Production of hybridoma growth factor by human monocytes. *Eur J Immunol* 1987; **17:** 1411.
8. Van Damme J, Van Beeumen J, Decock B, Van Snick J, De Ley M, Billiau A. Separation and comparison of two monokines with PAF activity (interleukin-1 beta and hybridoma growth factor): identification of leucocyte-derived HGF as interleukin-6. *J Immunol* 1988; **140:** 1534.
9. Van Damme J, Cayphas S, Opdenakker G, Billiau A, Van Snick J. Interleukin-1 and poly (ri) poly (rc) induce production of a hybridoma growth factor by human fibroblasts. *Eur J Immunol* 1987; **17:** 1.
10. Gauldie J, Richards C, Harnish D, Lansdrop P, Baumann H. Interferon β2, B-cell stimulatory factor type 2 share identity with monocyte-derived hepatocyte-stimulating factor and regulates the major acute phase protein response in liver cells. *Proc Natl Acad Sci USA* 1987; **84:** 7251.
11. Garman R, Jacobs K, Clark S, Raulet D. B-cell-stimulatory factor 2 ($\beta^2$ interferon) functions as a second signal for interleukin-2 production by mature murine T cells. *Proc Natl Acad Sci USA* 1987; **84:** 7629.
12. Lotz M, Jirik F, Kabouridis P, *et al.* B-cell stimulating factor 2, interleukin-6 is a costimulant for human thymocytes and T lymphocytes. *J Exp Med* 1988; **167:** 1253.
13. Ikebuehi K, Wong G, Clark S, Ihle J, Hirai Y, Ogawa M. Interleukin-6 enhancement of interleukin 3-dependent proliferation of multipotential hemopoietic progenitors. *Proc Natl Acad Sci USA* 1987; **84:** 9035.
14. Pignol B, Henane S, Mencia-Huerta JM. Effect of platelet-activating factor and its specific receptor antagonist, BN 52021 (ginkgolide B), on interleukin-1 (IL-1) release and synthesis by rat spleen adherent monocytes. In: Braquet P, ed. *The Ginkgolides, Vol 1.* 1988; 703–712.
15. Pignol B, Henane S, Sorlin B, Rola-Pleszczynski M, Mencia-Huerta JM, Braquet P. Effect of long term treatment with platelet-activating factor on IL-1 and IL-2 production by rat spleen cells. *J Immunol* 1990; **145:** 980.
16. Gery I, Waksman B. Potentiation of thymus derived lymphocyte response to mitogen. Part two: The cellular sources of potentiating mediators. *J Exp Med* 1972; **136:** 143.
17. Braquet P. Treatment or prevention of PAF-acether disorders provoked by a new series of highly specific inhibitors. GB Patent 84/ 18 424 (July 19, 1984). Belg. EE 901.015 (see CA 103. 189808 d).
18. Nijsten M, De Groot E, Ten Dius H, Klasen H, Hack C, Aarden L. Serum levels of interleukin-6 and acute phase responses. *Lancet* 1987; **ii:** 921.
19. Yoshimoto T, Nakanishi K, Hirose S, *et al.* High serum level reflects susceptible status of the host to endotoxin and IL-1/tumor necrosis factor. *J Immunol* 1992; **148:** 3596.
20. Kohase M, Heuriken-Destefana L, May P, Vilcek J, Sehgal P. Induction of beta 2 interferon by tumor necrosis factor: a homeostatic mechanism in the control of cell proliferation. *Cell* 1986; **45:** 659.
21. Kohase M, May T, Tamm I, Vilcek J, Sehgal P. A cytokine network in human diploid fibroblasts: interactions of beta-interferons, tumour necrosis factor, platelet-derived growth factor, and interleukin-1. *Mol Cell Biol* 1987; **7:** 273.

**Received 15 January 1994; accepted in revised form 31 March 1994**
